# *Helicobacter pylori* BabA–SabA Key Roles in the Adherence Phase: The Synergic Mechanism for Successful Colonization and Disease Development

**DOI:** 10.3390/toxins13070485

**Published:** 2021-07-13

**Authors:** Dalla Doohan, Yudith Annisa Ayu Rezkitha, Langgeng Agung Waskito, Yoshio Yamaoka, Muhammad Miftahussurur

**Affiliations:** 1 Faculty of Medicine, Universitas Airlangga, Surabaya 60131, Indonesia; doctordoohan@gmail.com (D.D.); langgengaw@gmail.com (L.A.W.); 2 Faculty of Medicine, Universitas Muhammadiyah Surabaya, Surabaya 60113, Indonesia; yudithannisaayu@gmail.com; 3 Department of Environmental and Preventive Medicine, Faculty of Medicine, Oita University, Yufu 879-5593, Japan; 4 Department of Medicine, Gastroenterology and Hepatology Section, Baylor College of Medicine, Houston, TX 77030, USA; 5 Division of Gastroentero-Hepatology, Department of Internal Medicine, Faculty of Medicine—Dr. Soetomo Teaching Hospital, Universitas Airlangga, Surabaya 60286, Indonesia; 6 Institute of Tropical Disease, Universitas Airlangga, Surabaya 60115, Indonesia

**Keywords:** BabA, SabA, adherence, infectious disease

## Abstract

*Helicobacter pylori* is a pathogenic microorganism that successfully inhabits the human stomach, colonizing it by producing several virulence factors responsible for preventing host self-defense mechanisms. The adherence mechanism to gastric mucosal tissue is one of the most important processes for effective colonization in the stomach. The blood group antigen-binding adhesion (BabA) and sialic acid-binding adherence (SabA) are two *H. pylori* outer membrane proteins able to interact with antigens in the gastroduodenal tract. *H. pylori* possesses several mechanisms to control the regulation of both BabA and SabA in either the transcriptional or translational level. BabA is believed to be the most important protein in the early infection phase due to its ability to interact with various Lewis antigens, whereas SabA interaction with sialylated Lewis antigens may prove important for the adherence process in the inflamed gastric mucosal tissue in the ongoing-infection phase. The adherence mechanisms of BabA and SabA allow *H. pylori* to anchor in the gastric mucosa and begin the colonization process.

## 1. Introduction

*Helicobacter pylori* is widely known as one of the pathogenic microorganisms that can successfully colonize the human stomach. Moreover, it is reported to infect half of the world’s population with regional variations [1]. *H. pylori* can withstand the harsh condition of the human stomach due to its complex mechanisms activated once it enters the stomach. With the possession of urease and sheathed flagella, *H. pylori* can withstand the highly acidic human stomach and actively move toward the gastric epithelial cell surface through the mucosal barrier. Therefore, *H. pylori* mostly resides in the mucus overlying the epithelium [2,3] and is less likely found in epithelial cells. However, an active moving mechanism alone cannot sufficiently promote *H. pylori* survival in a highly acidic stomach niche, as it must anchor itself to the human gastric epithelial cell membrane; otherwise, they would return into the stomach lumen and be wasted from the stomach. Once *H. pylori* have anchored to the host, they will remain within the stomach and continue to colonize this organ. Despite the high infection rate reported in various studies worldwide, most of the infected individuals will remain asymptomatic. However, studies reported that *H. pylori* is associated with the development of several gastroduodenal diseases, such as chronic gastritis, peptic ulcer disease, gastric cancer, and mucosa-associated lymphoid tissue (MALT) lymphoma [4,5,6].

The adherence mechanism is assumed to be a significant process for effective colonization of *H. pylori* in the stomach. In fact, the human stomach has a mechanism of continuous mucus production with a high turnover and rapid regeneration supporting the importance of *H. pylori’s* anchor mechanisms. To date, many studies have reported the importance of outer membrane proteins (OMPs) for the attachment to the gastric epithelial cells. Ever since the *H. pylori* whole-genome sequence has been published, dozens of OMP genes from various gene families have been hypothesized and investigated. Among them, the members of *H. pylori’s* outer membrane protein (Hop) group, the sialic acid-binding adherence (SabA/Omp17), and the blood group antigen-binding adhesion (BabA/Omp28) are some of the most frequently studied OMPs. This review will summarize our most current understanding on *H. pylori* BabA and SabA, including the regulation, adherence mechanism, pathogenic roles on disease development, and association with other virulence factors.

## 2. BabA: History, Gene, and Structure

BabA is part of the Hop family and also known as HopS, ranging from 75 kDa to 80 kDa in size and one of the earliest *H. pylori* adhesin found [7]. It is the most common and widely studied OMP, as compared to its paralogs, BabB and BabC. BabA was first discovered by observing the binding mechanism between *H. pylori* with labeled fucosylated blood group antigens [7]. Its localization on the bacterial surface was confirmed by probing with the Le^b^ antigen and then visualized using immunogold electron microscopy. The paralog *babA*, named *babB*, was also introduced in that study, and the 78 kDa-sized protein encoded by this paralog gene was introduced as BabB. The same study also examined the affinity of BabA and Le^b^ binding and showed that the adhesin-specific receptor complex was formed in an equilibrium condition. Further, they revealed that the affinity constant (*K*a) between BabA and Le^b^ was ~1 × 10^10^ M^−1^ [7]; however, this result was based on the interaction between whole *H. pylori* bacteria (strain CCUG 17875) and the Le^b^ antigen. Another study conducted by Hage et al. examined the binding capability of purified recombinant BabA proteins with the Le^b^ antigen and showed that the binding affinity between BabA–Leb was low [8].

The *H. pylori* genome encodes various types of OMPs, and most of them are expressed on the bacterial surface area. The *babA*, *babB*, and *babC* were identified based on the high product similarity (BabA, BabB, and BabC, respectively) to the Le^b^ antigen binding protein [7], especially in the *N*-terminus region. Another study also classified BabA and BabB proteins into a similar paralogous OMP family based on the significant similarity in *N*- and *C*-terminal regions [9]. However, sequence divergence exists in the mid-region, and phylogenetic analysis demonstrates a geographical clustering between strains [9]. The variation in the mid-region of *bab* gene indicates that this region might be important for encoding unique functions [10].

Within the *H. pylori* genome, *babA, babB, and babC* have been reported to be located in different loci between strains [11]. Marker genes, such as *s18*, the heme oxygenase gene (*hp0318*), and *hypD* were used to determine the chromosomal location of each *bab* gene [12]. In the *H. pylori* 26695 strain, Loci A, B, and C are located downstream of the *hypD*, *s18*, and heme oxygenase genes (hp0318), respectively [12]. In strain J99, *babA* and *babB* were located downstream of *hypD* and *s18*, respectively. However, the location of *babA* and *babB* was reversed in strain 26695. Interestingly, some strains may possess multiple copies of *babA*, and some strains even do not possess *babA*. Strains possessing multiple *babA* genes may have different locus location for each gene. For example, strain SouthAfrica7 has two *babA* genes located in loci A and B. Previous studies conducted in different countries showed that most strains have *babA* and *babB* located in loci A and B, respectively [12,13,14,15,16]. However, the *babA* gene might be located outside the well-characterized loci A, B, and C. A previous study by Hennig at al. revealed the presence of two *babA*-positive strains, but the chromosomal locus was indeterminate, suggesting the possibility that other chromosomal loci for *babA* are yet to be identified [14].

A previous study by Hage et al. revealed the crystal structure of BabA and analyzed the BabA–Le^b^ complex structure [8]. BabA contains two main α-helical regions named the handle and head region and β-sheet motif named the crown. BabA has a high similarity with SabA. However, unlike BabA, SabA lacks the four-strand antiparallel β-sheet crown which is important in Leb binding sites [8].

## 3. SabA: History, Gene, and Structure

SabA is part of the Hop family and also known as HopP. Compared to BabA, Sab proteins are smaller in size, with approximately 70 kDa. SabA is able to recognize and bind to sialylated glycans, mainly to sialylated Le^x^ [16]. Unlike BabA, SabA does not possess the crown β-sheet motif which, subsequently, results in SabA’s lack of affinity towards Le^b^ [8,17].

The *sabA* is a highly divergent gene and the regulation is complex. Goodwin et al. reported that *sabA* transcription is regulated through changes in dinucleotide repeats located in the 5′ of the *sabA*. The ArsRS signal transduction system has also been reported to take part in the *sabA* expression control. The combination of alternative transcriptional and expression mechanisms, such as slipped-strand mispairing replication and ArsRS, may be important for *H. pylori’s* capability to adapt with immune responses and highly-dynamic environment of the human stomach [18].

Mahdavi et al. firstly identified SabA using a “retagging” method and successfully recovered a 66 kDa-sized protein from the strain J99 [19]. Mass-spectrophotometry was then used to identify peptides. Four peptides were matched—peptide encoded by JHP662 in J99 (HP0725 in the 26695 strain). Additionally, two of those peptides were matches with another gene, JHP659 (HP0722 in 26695). Those genes were then substituted with an antibiotic-resistant cassette (*camR*) to confirm the gene that really expresses SabA. As a result, the sLe^x^ and sLe^a^ antigen binding activity was not found in the JHP662 mutant; however, the activity still existed in the JHP659 mutant [19], confirming that SabA was encoded by the JHP662 gene.

Another study by Pang et al. revealed the three-dimensional structure of *H. pylori* SabA by using X-ray crystallography [17]. The SabA protein was reported as a “club-shaped” molecule consisting of a number of features important for the interaction with host-cell glycoproteins. In the same study, they also showed that SabA is not only able to bind with sialyl-Le^x^, but also able to bind with the non-sialylated Le^x^ antigen, although with a weak affinity. SabA was shown to be a functional protein and capable of specifically binding to sialyl-Le^x^ and Le^x^ [17]. The *N*-terminal region of SabA contains important sites for sugar-binding activity, and this section consists of amino acid residues that are conserved between SabA and BabA, suggesting that the mutation of ortholog residues in BabA will also influence the BabA affinity towards the Le^b^ antigen.

## 4. Production and Regulation of BabA and SabA in Transcriptional and Translational Levels

*H. pylori* genomes can contain single or multiple copies of *babA*, and the region encoding this gene may be located in different loci depending on the strain. However, functional determination of the gene is also necessary. In general, two *babA* genotypes exist: *babA1* and *babA2*. *babA2* can encode a complete adhesin, whereas *babA1* is a defective gene due to the absence of the start codon sequence (ATG) and signal peptide [7]. The lack of the start codon in *babA1* resulted in translational failure due to the absence of an initiation signal. Bäckström et al. supported these data and reported that *babA1* is transcriptionally silent due to additional nucleotides within the −10 and −35 sites of *babA1*, and this addition leads to a reduced promotor region strength [20]. However, the *babA1* genotype is extremely rare, as it was only reported in strain CCUG17875. Most of the strains analyzed in various studies from different parts of the world only reported the presence of the *babA2* gene and the absence of *babA1*. However, a *babA2*-cam, a mutant strain derivative of CCUG17875, regained the binding ability with Le^b^ through a recombination process with the silent *babA1* into the expressed and partially homologous *babB* locus [20]. This *babB/A* chimera sequence analysis showed that the recombination involved a 66-bp patch after a sequence of a 47-bp *babB*-specific sequence in the 5′ ends. The exact reason of this phenomenon is unclear; however, it was hypothesized that this recombination might occur from gene conversion or from spontaneous DNA natural transformation from the lysis of other cells in the culture [20]. Strain NCTC11638 showed a recombination process similar to CCUG17875, involving the recombination of a silent *babA* into an expressed *babB* gene, forming a *babB/A* chimera [20]. Conversely, the recombination of *babB* into an expressed *babA* gene was also observed [9]. However, although not impossible, the occurrence of chimeric *babA* in the population level is expected to be low.

In vivo experiments showed that the loss of the BabA expression may occur and, subsequently, result in the loss of the ability to bind with the Lewis antigen in the rhesus macaques and Mongolian gerbil [21,22,23]. Several hypotheses have been raised with regard to this lost BabA expression. First, the change in the number of Cysteine–Threonine (CT) repeats in the 5′ ends, resulting in a frameshift mutation [7,15,21,22]. This slipped strand mispairing mechanism (SSM) was also found in at least five other *H. pylori* OMPs [18,24,25,26] and is believed to play a significant role in regulating the expression. Second, nucleotide substitution, deletion, or insertion occur in *babA*, resulting in a truncated and non-functional adhesin [23]. Third, Styer et al. showed a substitution of *babA* that expresses a Le^b^-binding site with a nonbinding allele. In this case, the replacement of six amino acids from *babA2* to *babA1* sufficiently eliminated the product’s binding ability to the Lewis B antigen [22]. Fourth, an in vivo study using C3H/HeJ mice, an animal model with a TLR4-signaling defect, showed the lost ability of BabA to bind with Lewis B; however, an existing BabA expression, suggesting the host innate immunity response, might be important [22]. Further, it is important to remember that animal models could not represent the actual human gastric environment. *H. pylori* is hypothesized to have the ability to modify BabA by changing the adhesin structure, successfully adapting to changes of the Lewis antigen or glycan expression at the gastric epithelial cell [27]. Therefore, BabA expression may vary in both the translational and transcriptional levels [9,15,20,21,22,23].

Upon the occurrence of *H. pylori* infection and gastric inflammation, the sialylated Lewis x (sLe^x^) is upregulated. The sLe^x^ then becomes the main target receptor for the SabA adhesin. Therefore, SabA’s binding ability may be important to strengthen the *H. pylori* adhesion to the gastric epithelial cells, especially in the ongoing *H. pylori* infection process. SabA is encoded by *sabA,* and the transcription level of *sabA* is affected by the salt concentration, where a high-salt level induces a higher SabA transcription level [28]. In addition, previous studies have reported that a high-salt level also promotes *H. pylori* pathogenicity by upregulating other OMP transcription levels, such as *hopA* and *hopQ*, as well as increasing other virulence factor transcription levels, such as *cagA* [28,29]. Therefore, *H. pylori* can still adapt with environmental changes and express various OMPs important for the adherence process. The functional state of SabA is also regulated by the slipped strand-mispairing mechanism and the number of cysteine–threonine dinucleotide repeats (CT repeats) in the 5′ region of *sabA*. The exact mechanism to trigger the switch between “switch On” (functional) and “switch Off” (non-functional) remains unclear. However, it is hypothesized that *H. pylori* can manage the switch depending on various environmental conditions. For example, the J99 isolate used by Mahdavi et al. to characterize the SabA properties possessed 10 CT repeats, whereas the *sabA* of J99 deposited in GenBank only possessed 9 CT repeats [19]. The “on/off” switch of the SabA expression suggests that SabA expression can rapidly respond to changing conditions in the stomach, possibly to prevent the host immune mechanism. Therefore, by maintaining the balance between functional and non-functional states, *H. pylori* might be able to survive in a highly dynamic environment of the human stomach. In addition, SabA expression might also be regulated by the gastric acid secretion [30]. Yamaoka et al. reported that the combination of “On” OMPs, including OipA, HopZ, SabA, and SabB, were responsible for the colonization ability in the mice model. Conversely, changes of two or more OMPs into “Off” might result in the loss of the ability to colonize the mice model, suggesting that the combination of several functional OMPs was more potent for the survival of *H. pylori* in the gastric environment [31]. 

## 5. BabA and SabA Interaction with Host Cell

During the discovery, BabA was found to possess a binding activity with the Le^b^ antigen [7]. Ever since, binding sites in BabA were found as a receptor for other antigens in the gastroduodenal tract. However, the most important characteristic of BabA is its ability to recognize the antigen from the ABO blood group antigens. The ability to bind with ABO group antigens then became the basis of BabA classification into “generalist” and “specialist” type. The “generalist” strains can bind with the group O antigen as well as groups A and B antigens. Meanwhile, the “specialist” strains can bind to the group O antigen only. However, due to the plasticity of *babA* and the ability to adjust the expression level, for example, by forming a chimeric *babA*, the “specialist” strains can broaden the spectrum of the binding phenotype and become a “generalist” [32]. The importance of BabA for the adhesion was also shown by Skindersoe et al. by quantifying the adhesion level of *H. pylori* with gastric cell lines, such as AGS and MKN45 [33]. Interestingly, the ability of *H. pylori* to form a chimeric BabA gene, such as *babA/B* or *babB/A*, might be important for the successful colonization in the gastroduodenal tract. These alterations served as mechanisms for better adaptation in the highly dynamic gastroduodenal tract environment, yielding a stronger and permanent host infection, rather than spontaneous elimination [34].

Those Lewis antigens are commonly found in several types of mucins and gastric epithelial cells. MUC1 and MUC5AC in the stomach and MUC5B in the salivary glands were reported to contain various types of Lewis antigens, subsequently making the oral cavity and stomach as potential locations for the BabA binding mechanism (Table 1). The *babA*-positive strains bound to the Le^b^ antigens from the gastric juice, saliva, healthy stomach, and gastritis stomach showed that strains with Le^b^-binding adhesion are more dependent on the blood group of the host than the anatomic site [35]. Mahdavi et al. also reported that sLe antigens were increased in a *H. pylori*-infected rhesus monkey and reported sialyl-dimeric-Lewis (sdiLe^x^) glycosphingolipid as a receptor for *H. pylori* [19].

**Figure 1:**
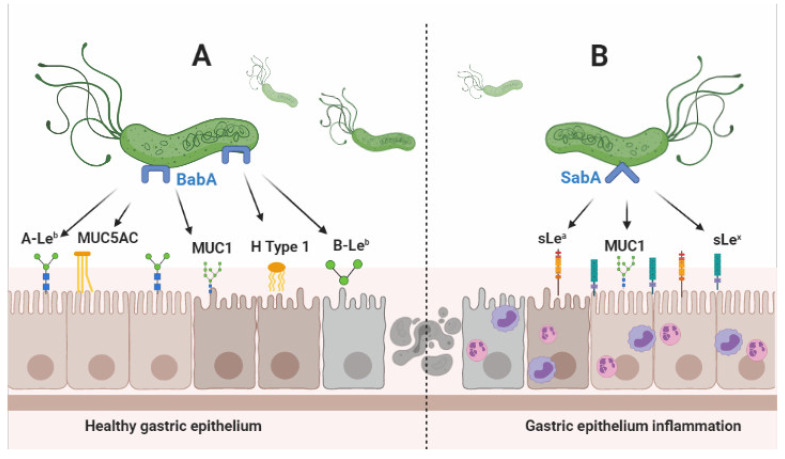
Schematic of *H. pylori’s* anchoring processes facilitated by BabA and SabA. (**A**) In a healthy gastric epithelium cell, BabA can bind with various antigens, such as the A/B-Le^b^, MUC5AC, MUC1, and H type 1 antigen. The ability of BabA to interact with various antigens shows its importance in the initial adherence process on the gastric epithelial cells. (**B**) sLe^x^ and sLe^a^ antigens are upregulated in inflamed gastric epithelium cells. Therefore, SabA might play a more important role for anchoring to epithelium cells with an ongoing inflammation process.

The ability to recognize sialylated glycans is a unique feature of SabA. Mucins produced in the salivary glands and stomach had the most diverse binding sites for *H. pylori*, including MUC5B, MUC7, MUC5AC, and salivary agglutinin [36]. The ability of SabA to bind sialylated Lewis antigens (sLe^a^ and sLe^x^) is important in *H. pylori* pathogenesis because *H. pylori* infection is associated with the replacement of non-sialylated Lewis antigens with sialylated one. To support this fact, a higher level of sialylated glycoproteins was found in *H. pylori*-infected patients, and the level was decreased after a successful eradication [37,38]. In healthy gastric mucosa, BabA plays a big role in adhesion; however, in gastritis, SabA’s ability to bind to the specific receptor increases due the higher level of sialylated glycans [33]. In the rhesus monkey model, a significantly increasing level of the sialylated Le antigen occurred as early as 1 week within inoculation; however, most of them returned to normal levels within 10 months, indicating that this mechanism might play a significant role in the early infection phase more than in chronic infection [39]. The sialylated Lewis x and SabA interaction is reported to be an important factor for *H. pylori* colonization in individuals without or with low Lewis x antigen expression [36]. The *sabA*-positive status was one of the important factors associated with an increased *H. pylori* density gastric mucosa and adherence ability in the mice model, together with OipA, SabB, and HopZ [31]. Figure 1 shows the schematic of *H. pylori*’s anchoring processes facilitated by BabA and SabA.

An animal model study by Falk et al. examined *H. pylori’s* ability to colonize a mice model. They confirmed that *H. pylori* is able to colonize FVB/N-transgenic mice expressing Le^b^ epitope by expressing BabA [40]. However, previous studies also reported that in various animal models, such as gerbil, rhesus macaques, and mice, the expression of functional BabA was lost during the infection phase [21,22,23]. This phenomenon might be related to the slipped-strand mechanism leading to an early stop codon and the recombination of *babA* and *babB*, resulting in the loss of BabA expression. However, the loss of BabA expression in the animal models shows that the expression of BabA adhesion is highly dynamic and similar to infection in humans; this phenomenon also might be related to *H. pylori’s* mechanism to avoid an unfavorable colonization environment and avoid host immune responses.

## 6. BabA, SabA, and Disease Development

To date, studies aiming to find the relationship between *babA* and disease development have been conducted in different parts of the world. However, the relationship between *babA2*-positive strains and the increased risk of clinical outcome development remains controversial [46,47]. Various studies have suggested the association between *babA2* and the increased gastric mucosal inflammation and the increased risk for clinical outcome development. A study by Fujimoto et al. showed that the BabA low-producer strains had more severe mucosal injury and were associated with gastritis [46]. This result showed the possibility that cellular injury is not merely dependent on Le^b^ binding activity. Another possibility is that the in vitro condition might not be able to represent the actual condition of the human stomach. Saberi et al. revealed that *H. pylori* strains that produced low levels of BabA and exhibited a lower Le^b^ binding activity, but not strains producing high levels of BabA with a high Le^b^ binding activity, were better adapted for colonization in gastric meta-plastic patches in the duodenum and, subsequently, enhanced duodenal ulcer risk [34]. In addition, a meta-analysis revealed that the combination of *babA2*-positive and *cagA*-positive *H. pylori* was significantly associated with duodenal ulcers in the Western population [48]. Although the exact reason of this phenomenon has not yet been fully established, the study implying that *babA2*-positive strains, which possess the Le^b^ binding activity, might be associated with other virulence factors and have a unique pathogenicity in duodenal ulcer development in the Western population. Other studies found a relationship between *babA2*-positive *H. pylori* strains with atrophic gastritis [46], peptic ulcer [34], and gastric cancer [49]. Sugimoto et al. examined the importance of BabA and OipA’s binding activity to inflammation levels in Mongolian gerbils. They showed that infection in *babA* and *oipA* mutants resulted in a decreased inflammatory cell infiltration as well as inflammatory cytokine levels [50]. However, in vitro or in vivo *studies* examining the binding activity between BabA and the Lewis antigen alone might inadequately conclude the direct role of BabA on disease development. BabA plays a major role in *H. pylori* colonization of the gastric niche. To significantly disturb host normal physiology, however, other virulence factors are likely required to play a contributory and potentially synergistic role with the function of BabA. Therefore, the binding activity of BabA might be important to directly or indirectly facilitates the translocation of other virulence factors into epithelial cells.

The importance of SabA in the development of gastroduodenal diseases has been investigated in many epidemiologic studies. SabA has been associated with atrophic gastritis, intestinal metaplasia, and gastric cancer [30]. However, the association between SabA and diseases in the gastroduodenal tract remains controversial. Upon *H. pylori*-induced gastritis, neutrophils and monocytes infiltrate into the gastric mucosa. SabA is essential for nonopsonic activation of neutrophils and, subsequently, specifically bind to neutrophils through the sialylated carbohydrates. Stimulated neutrophils will produce more reactive oxygen species (ROS) and cause oxidative stress and damages of the gastric epithelium cells [51,52], demonstrating SabA’s important role as a true virulence factor responsible not only for colonization, but also for inducing a pathologic opportunity, leading to disease development. Table 2 summarizes previous studies examining the association between BabA and SabA with disease.

## 7. Association with Other Virulence Factors

The role of BabA and SabA in adherence mechanisms is well characterized. However, their association with other virulence factors remains unclear. BabA and Le^b^ antigen binding might be important to efficiently activate the type four secretion system (T4SS) by subsequently enhancing the inflammation in the stomach [63]. It is possible that in the early infection phase, BabA binds with the antigens in stomach epithelial cells and supports the CagA translocation through a syringe-like mechanism of T4SS. CagA then induces cellular mechanisms within host cells. *H. pylori* was also found to induce DNA double-strand breaks through a BabA-dependent manner, a result supported by the decreased capability of the *babA* mutant to induce DNA double-strand breaks [64]. The presence of *cagA* was also associated with bacterial binding to the cell via the BabA-Le^b^ antigen binding. Ilver et al. showed that the binding activity was found in 73% (54 of 74) of CagA-positive strains, significantly >5% (1 of 20) of the CagA-negative strains tested [7].

*H. pylori* is reported to have various homologous genes with other non-*H. pylori* species, such as the gene responsible for urease production. Due to the important contribution of urease for acid resistance, this gene is available in several species in the *Helicobacter* genus, such as *H. pylori* and *H. mustelae* [65]. Although *H. pylori* has diverse genomes, various regions and genes are conserved among different *H. pylori* strains, but absent in non-*H. pylori* species in the *Helicobacter* genus, such as cag-pathogenicity island (cag-PAI), *bab*, and *sab* [66]. In addition, Hage et al. reported that there were no DNA or protein sequences with similarity to the crown or β-strand unit of *H. pylori* [8]. These data showed that BabA and SabA might be unique in *H. pylori* and may be considered as potential targets associated with *H. pylori’s* adaptive capability and pathogenicity in the human stomach.

## 8. Future Insights

Even though many studies demonstrated the importance of BabA and SabA for *H. pylori* attachment to the host, the importance of these OMPs to directly disturb a host’s normal physiology remains questionable. Further studies might be needed to prove BabA and SabA’s role as true virulence factors related to disease development. Moreover, the association between OMPs and other virulence factors remains an enigma. Further studies might be able to reveal whether BabA and SabA were directly mechanically associated with CaPAI, VacA, and HopQ.

Currently, *H. pylori* research mainly uses human gastric biopsy, fixed gastric tissue, cell lines, and animal models. However, currently, the use of human organoids has emerged as another option to conduct *H. pylori* study. Gastric organoids are a three-dimensional culture system grown from gastric stem cells and comprise the differentiated cell type of the human stomach [67]. Gastric organoids can be used as a model to investigate *H. pylori* pathogenesis. Nowadays, the use of gastric organoids to examine BabA and SabA pathogenesis is still limited. However, it is not impossible to utilize organoids to conduct basic and medical research to deepen the understanding of BabA and SabA pathogenesis in the future.

The significant roles of BabA in SabA for the successful colonization and persistent infection support the use of these adhesins for preventive therapy for *H. pylori* infection. BabA and SabA can be considered as potential candidates in vaccine developments [68,69,70]. A study by Bai et al. successfully purified recombinant BabA2 and was recognized in the serum of *babA2*-positive *H. pylori*-infected patients and Balb/c mice infected with the recombinant BabA. Therefore, BabA might serve as a potential antigen with sufficient immunogenicity to be used as a vaccine. Hage et al. confirmed that the expressed recombinant BabA protein was properly folded during expression, and the purified protein possessed binding activity to Le^b^ glycoconjugates [71]. Multiepitope vaccine is another type of vaccine that can be considered. Combination of several antigenic epitopes from *H. pylori* virulence factors might serve as a potential vaccine candidate. Using a bioinformatics approach, Urrutia-Baca et al. designed a multiepitope oral vaccine with 11 epitopes for pathogenicity and colonization, including BabA and SabA [70]. The vaccine was then modeled and validated to achieve the three-dimensional structure and further analyzed for other vaccine parameters. The designed vaccine was predicted as an antigenic, non-allergenic, and soluble protein with appropriate molecular weight [70]. However, such a study still needs in vitro and in vivo examinations to determine the efficacy before applying in humans. *H. pylori* vaccine candidates have been tested in various animal models; however, the results widely varied and were inconclusive. The main issue in developing a *H. pylori* vaccine relates to the considerably diverse *H. pylori* genomes from different patients. Therefore, the use of *babA* and *sabA* as a subunit vaccine may be challenging due to the labile nature of these genes, where transcription and translation levels can be affected by various mechanisms and environmental factors.

## 9. Conclusions

*H. pylori* adherence to gastric epithelial cells is an indispensable process for successful colonization and persistent infection. BabA and SabA are important OMPs responsible for “anchoring” *H. pylori*. BabA is important in the early adhesion phase by attaching with various antigens in the human stomach, most importantly with ABO group antigens. Meanwhile, SabA mostly binds with sialylated glycans, which is upregulated and abundantly present in inflamed cells. Therefore, *H. pylori*’s ability to initiate infection and maintain the chronic infection might be dependent on the balance of BabA and SabA regulation. OMP regulations by changing the number of CT repeats, slip strand mispairing, and chimeric formation might be the *H. pylori* mechanism to adapt with a highly dynamic, ever changing environment of the human stomach. The importance of BabA and SabA also supports the use of these virulence factors as a potential candidate for vaccine development.

## Figures and Tables

**Table 1:** The known receptors for BabA and SabA binding activity.

Adhesin	Reference	Receptor	Receptor Location
BabA	Ilver et al. [7], Bugaytsova et al. [41]	Lewis B antigen	Stomach
	Ilver et al. [7], Benktander et al. [42]	H type 1	Stomach
	Linden et al. [35]	MUC5AC	Stomach
	Linden et al. [43]	MUC1	Stomach
	Benktander et al. [42]	Globo H	Stomach
	Benktander et al. [42]	Globo A	Stomach
	Walz et al. [44]	MUC5B	Saliva
	Linden et al. [36]	MUC7	Saliva
SabA	Mahdavi et al. [19], Sheu et al. [45]	Sialylated Lewis x	Stomach
	Benktander et al. [42]	Sialyl-neolactohexaosylceramide	Stomach
	Benktander et al. [42]	Sialyl-neolactooctaosylceramide	Stomach
	Aspholm-Hurtig et al. [32]	Sialylated Lewis a	Stomach
	Aspholm-Hurtig et al. [32]	Sialyl-lactosamine	Stomach
	Linden et al. [43]	MUC1	Stomach
	Walz et al. [44]	MUC5B	Saliva

**Table 2:** Studies examining the association between BabA and SabA with disease.

Adhesin	Published Year	Country	Total Sample	Association with Disease	Reference
BabA	2001	Korea	41	No	Kim et al. [47]
	2001	Italy	167	Yes, with diffuse gastritis, peptic ulcer, and duodenitis	Zambon et al. [53]
	2004	China	141	Yes, with duodenal ulcer	Han et al. [54]
	2006	Turkey	93	Yes, with gastric cancer	Erzin et al. [55]
	2010	Costa Rica	95	Yes, with atrophic gastritis	Con et al. [56]
	2010	Japan	95	No	Con et al. [56]
	2011	Iran	160	Yes, with gastric cancer	Talebi Bezmin Abadi et al. [49]
	2017	Bhutan, Myanmar, Nepal, Bangladesh	903	Yes, with peptic ulcer disease	Ansari et al. [13]
	2017	Iran	100	No	Sohrabi et al. [57]
SabA	2004	Netherlands	96	No	de Jonge et al. [58]
	2006	Colombia and USA	200	Yes, with intestinal metaplasia and atrophic gastritis	Yamaoka et al. [30]
	2006	Taiwan	145	No	Sheu et al. [59]
	2007	Japan	108	No	Yanai et al. [60]
	2013	Iran	120	No	Pakbaz et al. [61]
	2017	Japan	4	Yes, with iron deficiency anemia	Kato et al. [62]

## Data Availability

Not applicable.
